# Over-Expression of Meteorin Drives Gliogenesis Following Striatal Injury

**DOI:** 10.3389/fncel.2016.00177

**Published:** 2016-07-05

**Authors:** Jordan L. Wright, Charlotte M. Ermine, Jesper R. Jørgensen, Clare L. Parish, Lachlan H. Thompson

**Affiliations:** ^1^The Florey Institute of Neuroscience and Mental Health, The University of Melbourne, Melbourne, VICAustralia; ^2^NsGene, BallerupDenmark

**Keywords:** forebrain injury, striatum, neurogenesis, oligodendrogenesis, GDNF, brain repair

## Abstract

A number of studies have shown that damage to brain structures adjacent to neurogenic regions can result in migration of new neurons from neurogenic zones into the damaged tissue. The number of differentiated neurons that survive is low, however, and this has led to the idea that the introduction of extrinsic signaling factors, particularly neurotrophic proteins, may augment the neurogenic response to a level that would be therapeutically relevant. Here we report on the impact of the relatively newly described neurotrophic factor, Meteorin, when over-expressed in the striatum following excitotoxic injury. Birth-dating studies using bromo-deoxy-uridine (BrdU) showed that Meteorin did not enhance injury-induced striatal neurogenesis but significantly increased the proportion of new cells with astroglial and oligodendroglial features. As a basis for comparison we found under the same conditions, glial derived neurotrophic factor significantly enhanced neurogenesis but did not effect gliogenesis. The results highlight the specificity of action of different neurotrophic factors in modulating the proliferative response to injury. Meteorin may be an interesting candidate in pathological settings involving damage to white matter, for example after stroke or neonatal brain injury.

## Introduction

Meteorin is a member of a newly described family of secreted proteins that also includes the related protein, Meteorin-like. It is highly expressed in the mammalian brain, throughout mid- to late-development and is maintained at lower levels in the post-natal brain (Jorgensen et al., 2009). In adult rodents, Meteorin protein is most conspicuously expressed by Bergmann glia in the cerebellum, but also more diffusely at lower levels in glia cells throughout the brain. It was first characterized *in vitro* for its ability to promote astrocyte differentiation and axon growth in neurosphere cultures and dorsal root ganglion explants, respectively, (Nishino et al., 2004). Later studies established that Meteorin’s pro-gliogenic functionality acted on the Jak-STAT3 pathway (Lee et al., 2010; Sun et al., 2014), although to date, the receptor it functions through remains unknown.

*In vivo* studies in rodents demonstrated that after excitotoxic injury to the striatum, endogenous Meteorin is up-regulated in striatal s100β^+^ astrocytes (Jorgensen et al., 2011), and has also been observed in neurons after ischaemic damage (Wang et al., 2012). To explore it’s functional impact in this setting, subsequent studies demonstrated that over-expression of Meteorin via viral (Jorgensen et al., 2011) or encapsulated cell (Tornoe et al., 2012) delivery prior to excitotoxic injury resulted in significantly reduced levels of striatal damage (Jorgensen et al., 2011; Tornoe et al., 2012). More recently, studies have also begun to investigate the possibility that, in addition to neuroprotection of existing circuitry, Meteorin might also act as a survival factor for newborn neurons generated in response to striatal injury (Wang et al., 2012).

A number of studies have shown that, following striatal injury resulting in extensive cell loss, neuroblasts constitutively generated in the sub-ventricular zone (SVZ) can migrate into the damaged striatal parenchyma and differentiate into mature neurons (Arvidsson et al., 2001, 2002; Jin et al., 2001, 2003, 2006; Parent et al., 2002). The overall survival of these new neurons is notably poor and well below levels likely to be required for therapeutic replacement of neurons lost to the injury. This has motivated studies that investigate strategies to improve the survival of newborn neurons, for example through delivery of trophic factors. Topical delivery of soluble proteins known to influence cell survival, neural stem cell proliferation and neurogenesis in other disease models have proven to be robust regulators of these cellular processes in the context of forebrain injury. Examples of such factors are Glia-derived neurotrophic factor (GDNF; Kobayashi et al., 2006), Vascular endothelial growth factor (VEGF; Sun et al., 2003), along with Noggin and Brain-derived neurotrophic factor (BDNF; Benraiss et al., 2012) which have all been shown to positively regulate neurogenesis in the damaged striatum. The expression of Meteorin during neural development and in the adult striatum makes it an interesting candidate in this context. Recently, Wang et al. (2012) demonstrated that chronic infusion of Meteorin increased SVZ proliferation and neuroblast migration into the damaged striatum after medial cerebral artery occlusion in rats, resulting in increased numbers of surviving newborn neurons.

Here we sought to characterize the phenotype of new cells generated in the presence of Meteorin after striatal damage – including the identity of neuronal and glial subtypes. Following excitotoxic damage, we found that stable expression of Meteorin using a lenti-virus resulted in a small increase in the survival of new neurons which did not reach statistical significance. To contextualize this impact on neuronal survival, we also present results from GDNF expression in the same experimental paradigm, where there was a much larger effect on neurogenesis in response to injury. The major impact of Meteorin was in fact to drive gliogenesis, where birth-dating studies using Bromodeoxyuridine (BrdU) showed that there was a significant increase in the proportion of new s100β^+^ astrocytes and Oligodendrocyte transcription factor 2 (Olig2^+^) oligodendrocyte progenitors in response to damage to the adult striatum.

## Materials and Methods

### Ethical Approval

All experimental procedures carried out in this study conform to the rules set out by the Australian National Health and Medical Research Council published code of practice and experiments were approved by the Florey Neuroscience Institutes animal ethics committee (no. 09-105).

### *In Vivo* Procedures

Adult Sprague-Dawley rats were housed under a 12-h light/dark cycle with ad libitum access to food and water. Surgery was performed at 6 weeks of age using age matched animals. Prior to surgery, rats were deeply anesthetized with isoflurane (5% at 1 L/min) and kept under anesthesia (2-3% at 1 L/min) for the duration of surgery.

All surgical procedures were performed using a stereotaxic frame (Kopf, Germany) and intra-striatal delivery was performed using a fine glass capillary fitted to a 5 μl microsyringe (SGE Analytical Sciences, Australia). Under deep anesthesia, all animals received 2 μl of quinolinic acid (QA) solution (100 nmol/μl in 0.9% saline) injected over 2 min into the striatum (1.2 mm anterior and 3 mm lateral to bregma and 4 mm below the dural surface). The cannula was kept in place for 5 min after injection to minimize back-flow.

Twenty four hours post-injection, all animals underwent a second round of surgery (setup as previously described) for intra-striatal lentivirus delivery constructed as previously described (Jorgensen et al., 2011). Three treatment groups were compared within this study. Animals received one of either lentivirus encoding Meteorin (Jorgensen et al., 2011), GDNF (pHsCXW, NsGene), and GFP (pHsCXW, NsGene) for control animals via a 1.5 μl solution (1.2 × 10^5^ transforming units total) over 2 min into the striatum (1.2 mm anterior and 3.2 mm lateral to bregma and 4 mm below the dural surface). All vectors were under the control of the woodchuck post-regulatory element for constitutive transgene expression.

To label dividing cells, animals received BrdU (50 mg/kg i.p. using a 20 mg/ml solution in 0.9% saline) every 12 h for 2 weeks as previously described (Arvidsson et al., 2002), beginning the day of lentiviral delivery. Animals were perfused for histological assessment 4 weeks after BrdU treatment (6 weeks after QA lesion).

### Tissue Preparation

At 6 weeks after surgery, animals under deep anesthesia induced with isoflurane (5% at 1 L/min), subsequently received a lethal dose of pentobarbitone. Animals were then transcardially perfused with 50 ml saline (0.9% w/v) followed by 200–250 ml paraformaldehyde (PFA; 4% w/v in 0.1 M PBS). The brains were removed, post-fixed a further 2 h in PFA and cryoprotected in sucrose (20% w/v in 0.1 M PBS). Brains were sectioned in the coronal plane in a 1:12 series at a thickness of 30 μm on a freezing microtome (Leica, Germany).

### Immunohistochemistry

Primary antibodies were diluted in a solution of 0.1 M PBS containing 5% normal serum and 0.5% Triton X-100 (Amereso, USA) and incubated with sections overnight at room temperature. Prior to the addition of the secondary antibody solution, the sections were blocked with 5% normal serum for 0.5-1 h. Secondary antibodies were diluted in a solution of 0.1 M PBS containing 2% normal serum and 0.5% Triton X-100 and incubated for 2 h at room temperature. For fluorescent analysis, secondary antibodies were conjugated to Dylight Fluorophores 488, 549, or 647 and slide-mounted sections were cover-slipped with fluorescent mounting medium (DAKO, USA). For chromogenic analysis, secondary antibodies were conjugated to biotin and visualized using the Avidin/Biotin enzyme complex (VECTASTAIN ABC system, Vector Labs) coupled with peroxidase-driven precipitation of diaminobenzidine (DAB). DAB-labeled sections were dehydrated in alcohol and xylene, and cover-slipped with DePex mounting medium (BDH Chemicals, UK). For BrdU labeling, free-floating sections were incubated for 2 h at 65°C in 50% de-ionized formamide in 0.1 M PBS. The tissue was rinsed 3 × 5 min washes in 0.1 M PBS and acid treated with 2 M HCL for 30 min at 37°C. The tissue was then washed in 0.1 M Sodium Borate (pH 8.5) for 15 min and finally rinsed with 3 × 5 min washes in 0.1 M PBS.

Primary antibodies and dilution factors were as follows: mouse anti-APC (1:200; #OPT80, Calbiochem, Germany), rat anti-BrdU (1:300; #OBT0030, Axyll Laboratories, USA), mouse anti-Calretinin (1:1000; #7697, Swant, Switzerland), rabbit anti-Darpp32 (1:500; #AB10518, Millipore, Germany), goat anti-GDNF (1:200; #AB212NA, R&D, USA), chicken anti-GFP (1:1000; AB13970, AbCam, USA), rabbit anti-GFP (1:20,000; #AB13970, AbCam, USA), rabbit anti-Iba1 (1:200; #01919741, Wako, Japan), goat anti-Meteorin (1:400; #AF3475, R&D, USA), mouse anti-NeuN (1:200; #MAB377, Millipore, USA), rabbit anti-Olig2 (1:200; #AB9610, Millipore, USA), rabbit anti-s100β (1:200; #HPA015768, Sigma-Aldrich, USA).

### Imaging

Images of representative co-labeled cells were acquired via 40× or 63× PL-APO oil immersion objectives (Zeiss) using a Zeiss 780 laser-scanning confocal upright microscope. Representative overview images of DAB stained sections were acquired on a Leica DM6000B microscope using a HCX PL 5×/0.5 objective and tiled images aligned using Leica application suite v3.8 software. NeuN, BrdU, Iba1, Meteorin, GDNF and GFP immuno-labeled DAB representative images were acquired on the same microscope using HCX PL 20×/0.5 and HCX FL PLAN 40×/0.65 objectives (Leica). The brightness and contrast of individual images was adjusted using the photo enhancing software Photoshop v7.0 (Adobe) to optimally represent the immunohistochemistry observed through the microscope.

### Stereology and BrdU^+^ Cell Density Estimation

Stereological estimation was used to determine the number of BrdU^+^ cells residing in the anterior head of the striatum across the three treatment groups. For all animals, 30 μm coronal sections each 360 μm apart (1:12 series) were immuno-labeled for BrdU. To establish the region of interest, the rostral boundary of striatum was defined at 1.70 mm anterior to bregma and the caudal boundary defined at 0.26 mm anterior to bregma (spanning 0-1440 μm of the anterior striatum) and delineated by standard anatomical boundaries (Paxinos and Watson, 1998). Estimates of BrdU-labeled cells were determined using a fractionator sampling design according to optical dissector rules (Gundersen et al., 1988; Mayhew, 1991). Counting frame grid dimensions and fractionator x,y coordinates were determined using the grid overlay program Stereoinvestigator v7.0 (MicroBrightField, Williston, VT, USA, used on a microscope, Leica, with a 40× objective, Zeiss). Guard zones were set at 1 μm (top and bottom) and BrdU-labeled nuclei quantified within the counting frame (dimensions used were 90 μm × 90 μm) at periodic intervals (x = 300 μm, y = 350 μm) in the delineated region of interest.

To determine BrdU density, volumetric analysis was conducted to determine the volume of measured striatum. Striatal volumetric measurements for each brain were achieved via Cavalieri estimation using the program Stereoinvestigator v7.0 (MicroBrightField, Williston, VT, USA). The resultant volume was used to determine the BrdU^+^ cell density in the striatum based on the stereological estimation of BrdU^+^ cells and reported as BrdU^+^/mm^3^. The accuracy of the stereological estimations was determined by the coefficients of error and coefficients of variance. Estimations were deemed acceptable if coefficients were >0.1 (West et al., 1991).

### Cell Quantification

In order to assess the phenotype of newborn cells post-lesion in the striatum, sections from animals sacrificed at 6 weeks post-lesion were double or triple labeled using fluorescent immunohistochemical techniques. Sections were stained with either BrdU/NeuN/Darpp32, BrdU/NeuN/Calretinin, BrdU/s100β or BrdU/Olig2/APC. Due to the relatively low number of double and triple labeled cells in the striatum, cells were manually counted across 3 sections spanning the anterior head of the striatum (1.70, 1.34, and 0.98 mm anterior to bregma). An additional rostral striatal section was analyzed at 0.62 mm from bregma for quantification of BrdU^+^/s100β^+^ co-localization.

Using a confocal microscope (Zeiss) and a 20×/0.8 PL-APO objective (Zeiss), z-stacking, and x-y tile scanning features were used to capture large 3-dimensional volumes of the entire striatum for each tissue section. ZEN digital imaging software (Zeiss) was then used to unambiguously identify all double/triple labeled BrdU^+^ cells. When delineating the striatal volume for analysis, we did not include the highly damaged area immediately in the vicinity of the QA delivery, which we found to have a high level of brightly fluorescent non-specifically labeled cells. All BrdU^+^ cells initially identified as co-labeled, were verified by inspection with a 63× PL-APO oil immersion objective (Zeiss) and orthogonal reconstruction on the z-axis.

The total number of cells counted across the quantified striatum (1.70 mm–0.98 mm anterior to bregma) was estimated by extrapolation based on the series interval (1:12) and the number of sections counted (3) across the series. In addition to reporting total cell counts, total cell counts were normalized to BrdU^+^ cell density to account for any variability in these parameters across animals. This normalized result is reported in the text as a percentage of BrdU^+^ cell density (BrdU^+^/mm^3^).

For newborn Iba1^+^ microglia quantification, sections were stained with BrdU/Iba1. Due to the abundance of BrdU^+^/Iba1^+^ cells, one section per animal across treatment groups (*n* = 3 per group) was used for quantification (1.34 mm anterior to bregma). Striatal sections were systematically analyzed from the lateral ventricle to the site of injury across all animals for consistency. BrdU^+^ cells and BrdU^+^/Iba1^+^ cells were quantified in this manner. The resultant BrdU^+^/Iba1^+^ cell counts were normalized to the BrdU^+^ cell counts obtained from the same experiment and represented as a percentage.

### Statistics

Statistical analysis was conducted using Graphpad Prism v6.0 software. BrdU^+^ cell density, total cell counts and normalized cell counts were compared across the three experimental treatment groups using one-way ANOVA with *post hoc* correction (Bonferroni) for multiple comparison. In this study the data across experimental treatment groups is reported as the mean ± SEM.

## Results

### Neuronal Cell Loss and Local Proliferation after Excitotoxic Lesioning

**FIGURE 1:**
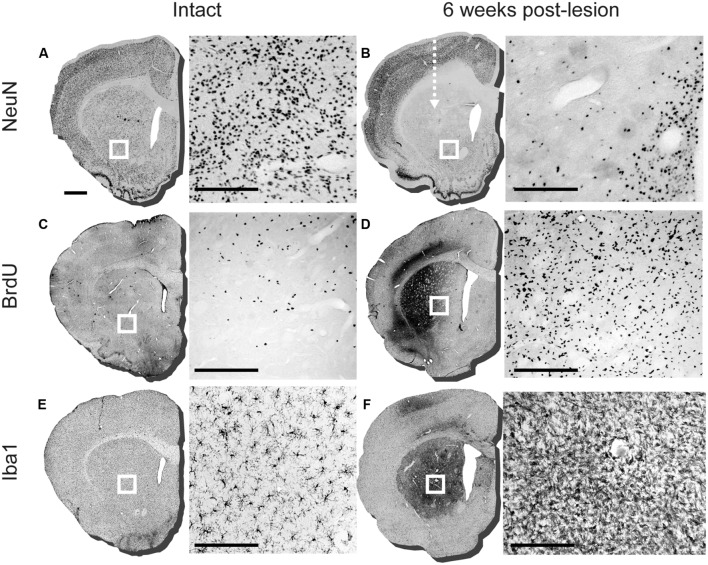
**Assessment of lesioning, proliferation, and microglial response 6 weeks after intra-striatal injection of quinolinic acid (QA).** Representative striatal sections of intact and lesioned brains 6 weeks post-lesion (arrow indicates injection site of QA lesion). The boxed area on each section corresponds to the adjacent 20× image detailing the immuno-labeling. **(A)** NeuN labeling in intact striatum and **(B)** 6 weeks post-lesion, detailing loss of neurons in adjacent 20× image. **(C)** BrdU labeling of an intact adult striatal section and **(D)** 6 weeks post lesion. **(E)** Iba1 staining in intact sections with adjacent 20× image detailing ramified microglia in the striatum and at **(F)** 6 weeks post-lesion with reactive microglia illustrated in the adjacent 20× image. Scale bar: 1 mm brain sections **(A-F)**, 200 μm boxed images **(A-F)**.

Immunohistochemical labeling of the mature neuronal marker NeuN showed that injection of QA into the anterior head of the striatum resulted in a robust loss of neurons (**Figures 1A,B**). In some cases we also observed neuronal loss in the overlying cortex, likely resulting from reflux along the injection tract (not shown). Labeling for BrdU showed a robust proliferative response during the first 2 weeks after the lesion (**Figures 1C,D**). The BrdU^+^ cells were distributed densely in the immediate vicinity of the QA injection site, along with what appeared to be some degree of non-specific labeling, and also more diffusely throughout the entire striatum. The excitotoxic lesion also resulted in an inflammatory response that was persistent at 6 weeks, as demonstrated by a large increase in the density of striatal Iba1^+^ microglia with a reactive, amoeboid morphology (**Figures 1E,F**). Some degree of microglial activation was also observed along the needle tract in the overlying cortex. Anatomically, the distribution of microglial activation matched well with the area of NeuN^+^ cell loss and increased BrdU^+^ labeling.

### Meteorin Is Highly Diffusible Within the Striatum When Over-Expressed Using a Lentiviral Vector

**FIGURE 2:**
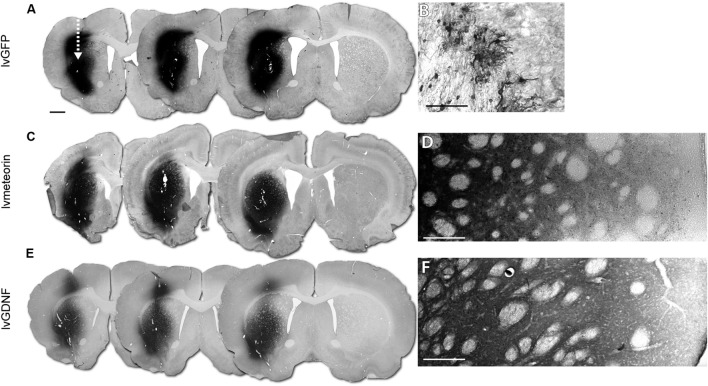
**Validation of lentiviral expression 6 weeks post-lesion.** Immunohistochemical analysis at 6 weeks post-injury in animals administered corresponding lentiviral over-expression constructs across three representative sections (arrow indicates injection site of lentivirus) **(A,C,E)** corresponding to 1.70, 1.34 and 0.98 mm from bregma, left to right. **(A)** lvGFP treated animals with GFP immuno-labeling with **(B)** 40× representative image of GFP^+^ astrocytic cells in the striatum. **(C)** lvMeteorin and **(E)** lvGDNF treated animals with Meteorin and GDNF immuno-labeling, respectively. **(D)** Tiled 40× image of lvMeteorin treated animals and **(F)** lvGDNF treated animals illustrate diffuse labeling within the striatum. Scale bar: 1 mm **(A)** analogous for **(C,E)**, 0.1 mm **(B,D,F)**.

Transduction of the striatum using lentiviral vectors carrying either GFP (lvGFP), GDNF (lvGDNF) or Meteorin (lvMeteorin) resulted in robust expression of the transgenes (**Figures 2A–F**). At 6 weeks, immunohistochemical analysis showed the expected cytoplasmic pattern of GFP expression (**Figure 2B**). The morphology of the GFP^+^ cells showed that predominantly astrocytes were transduced with the vector, although we observed a smaller population of GFP^+^ neurons also. Injection of either lvMeteorin or lvGDNF resulted in a robust expression throughout the entire striatum (**Figures 2C,E**). In both cases, the profile of expression was consistent with that expected for a secreted protein, including a diffuse labeling pattern that reduced in intensity with distance from the injection site (**Figures 2D,F**).

### Over-Expression of Meteorin Did Not Increase the Number of New Neurons in the Striatum after Excitotoxic Damage

**FIGURE 3:**
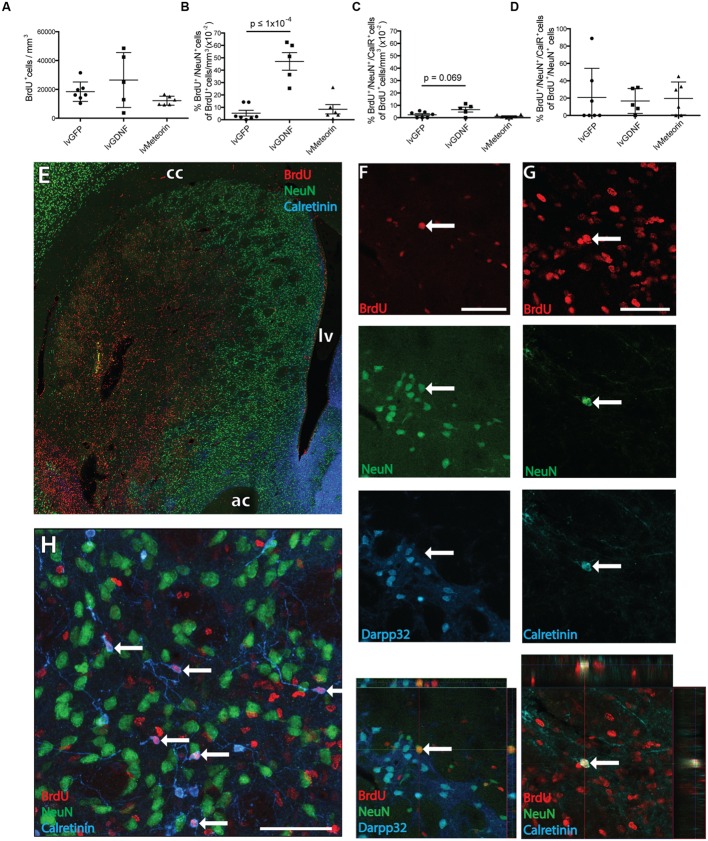
**Birth-dating of striatal neurons after quinolinic acid lesion to assess the effect of GDNF and Meteorin on these populations. (A)** Quantification of BrdU^+^ cell density across treatment groups revealed no significant change in newborn cells in the striatum. **(B)** Quantification of BrdU^+^ cells co-expressing NeuN across treatment groups normalized to BrdU^+^ cell density revealed lvGDNF increased the number of new neurons in the ipsilateral striatum, with lvMeteorin having no effect compared to lvGFP controls. **(C)** Quantification of BrdU^+^/NeuN^+^/CalR^+^ cells normalized to BrdU^+^ cell density identified lvGDNF treatment trended toward an increase in this population. **(D)** CalR^+^ cells normalized to total BrdU^+^/NeuN^+^ cells indicated differentiation into this phenotype is altered across all newborn neurons. Overview image of BrdU/NeuN/CalR staining **(E)** and representative orthogonal z-stack confocal images of immuno-labeled **(F)** BrdU^+^/NeuN^+^/Darpp32^-^ neurons and **(G)** BrdU^+^/NeuN^+^/CalR^+^ interneurons (arrow). **(H)** NeuN negative interneurons were also observed as a primary subset of BrdU^+^/Calretinin^+^ cells (arrows). Data represents mean ± SEM. lvGFP *n* = 7; lvMeteorin *n* = 6; lvGDNF *n* = 5. Scale bars: 100 μm. cc, corpus callosum; lv, lateral ventricle; ac, anterior commissure; CalR, Calretinin.

To assess the impact of Meteorin on neurogenesis in response to injury, animals were administered BrdU twice daily for 2 weeks to label newborn cells immediately after excitotoxic damage to the striatum. Animals were perfused for histology 4 weeks later to allow for cell migration and differentiation of BrdU-labeled cells. Stereological estimation of the total number of newborn BrdU^+^ cells in the striatum 6 weeks after injury showed no significant difference in the density of BrdU^+^ cells in the striatum between the lvGFP control (1.8 ± 0.3 x 10^4^ BrdU^+^ cells/mm^3^), lvMeteorin (1.2 ± 0.1 × 10^4^ BrdU^+^ cells/mm^3^) and lvGDNF (2.7 ± 0.9 × 10^4^ BrdU^+^ cells/mm^3^) groups (*p* = 0.67, lvGFP v lvGDNF; *p* = 0.95, lvGFP v lvMeteorin, one-way ANOVA, Bonferroni *post hoc* — **Figure 3A**), although the number of BrdU^+^ cells was notably variable in the lvGDNF group.

To estimate the number of new neurons generated during the 2 week BrdU-labeling period after striatal injury, cells unambiguously immuno-labeled for both BrdU and NeuN were counted in three coronal sections from each animal 4 weeks after the final BrdU injection (6 weeks after injury). This involved a first screening whereby every BrdU^+^ cell was inspected for evidence of double labeling, followed by a second level of screening where double labeling was confirmed by confocal analysis, including orthogonal reconstruction on the z-axis (**Figures 3E,F**).

Comparison of the total BrdU^+^/NeuN^+^ cell counts revealed that the lvGFP control group (86.6 ± 48.4 cells) was not significantly different in newborn mature neurons compared to lvMeteorin treated animals (88.5 ± 32.6 cells), but a significant increase was detected in the lvGDNF group (696 ± 116.2 cells, *p* = <1 × 10^-4^, one-way ANOVA, Bonferroni *post hoc*). To account for variability in BrdU dosage and striatal volume between animals and to assess the proportion of newborn cells within the striatum acquiring a mature neuronal phenotype, we normalized the total number of BrdU^+^/NeuN^+^ cells to BrdU^+^ cell density (BrdU^+^ cells/mm^3^) to acquire a percentage (**Figure 3B**). In lvGFP-treated controls a low percentage of newborn cells post-lesion obtained a NeuN^+^ phenotype (5.3 ± 0.2 × 10^-2^ % of BrdU^+^ cells/mm^3^). This was not significantly different to lvMeteorin-treated animals (8.5 ± 0.4 × 10^-2^ % of BrdU^+^ cells/mm^3^) but in the lvGDNF-treated group, an 11-fold increase in the percentage of BrdU^+^/NeuN^+^ cells was detected (47.1 ± 0.7 × 10^-2^% of BrdU^+^ cells/mm^3^, *p* = < 1 × 10^-4^, one-way ANOVA, Bonferroni *post hoc*; see **Figures 3E,F** for examples of immunohistochemistry).

Immunohistochemical co-labeling of BrdU^+^/NeuN^+^ cells with markers for striatal projection neurons (Darpp32) or certain interneuron subtypes (Calretinin) allowed us to assess the impact of Meteorin or GDNF over-expression on the phenotype of newly generated neurons following injury. Although previous studies have indicated a proportion of new neurons generated after injury acquire a Darpp32^+^ identity at (Arvidsson et al., 2002; Parent et al., 2002), we did not observe any Darpp32^+^/BrdU^+^ cells across the three treatment groups. Some of the BrdU^+^/NeuN^+^ cells were found to express Calretinin. Looking at BrdU^+^/NeuN^+^/Calretinin^+^ cells as a percentage of total BrdU^+^ cells/mm^3^, there was no significant difference across the three treatment groups although there was a trend toward a greater proportion in the lvGDNF group (6.5 ± 0.2 × 10^-2^ % of BrdU^+^ cells/mm^3^) compared to the lvGFP control group (2.4 ± 0.1 × 10^-2^ % of BrdU^+^ cells/mm^3^; *p* = 0.069, one-way ANOVA, Bonferroni *post hoc*) – **Figures 3C,G**. Similarly, as a percentage of total newborn neurons (BrdU^+^/NeuN^+^ cells) we observed similar proportions of cells obtaining Calretinin^+^ phenotype between the lvGFP (20.8 ± 12.7% of cells), lvMeteorin (19.6 ± 7.7% of cells) and lvGDNF groups (16.7 ± 6.4% of cells) – **Figure 3D**. We also observed across all groups numerous examples of BrdU+/Calretinin+ cells with aspiny neurites that did not express NeuN within the damaged striatum (**Figure 3H**). This may be indicative of a more immature population of newborn interneurons.

### Meteorin Increases Gliogenesis Following Striatal Damage

Co-labeling of BrdU^+^ cells with s100β or Olig2 showed that Meteorin over-expression significantly increased the proportion of BrdU^+^ cells with astrocyte and oligodendroglial progenitor phenotypes, respectively.

**FIGURE 4:**
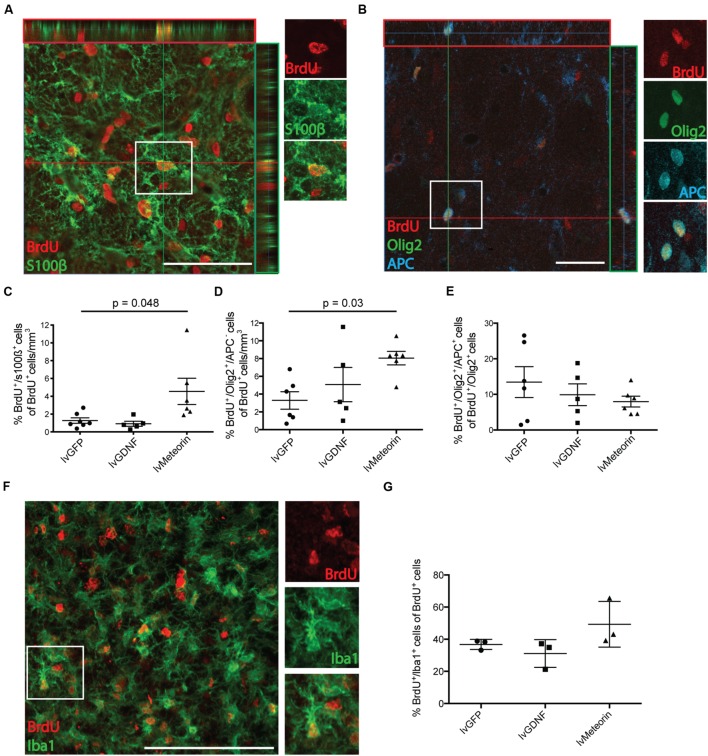
**Birth-dating of glial cells in the striatum after QA lesion and assessment of the effect of Meteorin on these populations. (A)** Confocal orthogonal representation of cells in the striatum ipsilateral to lentiviral delivery and lesion confirm double labeling of BrdU^+^ cells co-stained for either s100β to identify new born astrocytes or **(B)** Olig2 and APC to identify newborn OPCs (BrdU^+^/Olig2^+^/APC^-^ cells) and mature oligodendrocytes (BrdU^+^/Olig2^+^/APC^+^ cells; boxed insets detail double and triple labeling of the cells). Newborn glial cells were quantified and represented as a percentage of total BrdU^+^ cell density. **(C)** Cell quantification of BrdU^+^/s100β^+^ cells normalized to BrdU^+^ cell density revealed lvMeteorin-treated animals had significantly more newborn s100β^+^ astrocytes in the ipsilateral striatum compared to lvGFP controls. For Oligodendroglial lineages, **(D)** BrdU^+^/Olig2^+^/APC^-^ (OPCs) revealed a significant increase in this population in lvMeteorin-treated animals compared to lvGFP-treated controls. **(E)** The percentage of BrdU^+^/Olig2^+^ cells that express APC (mature OLs) was assessed and was not significant between treatment groups. Oligodendrocyte lineage cell quantification was normalized to BrdU^+^ cell density. **(F)** Representative BrdU/Iba1 stain with example BrdU^+^/Iba1^+^ cell (boxed) and **(G)** quantification of BrdU+/Iba1+ cells as a percentage of total BrdU showed no significant difference between treatment groups. Data represents mean ± SEM, lvGFP *n* = 7; lvMeteorin *n* = 6; lvGDNF *n* = 5 for astrocyte and oligodendrocyte quantification and *n* = 3 for microglia cell counts across all groups. Scale bar: 100 μm. OPC, Oligodendrocyte precursor cell; OL, Oligodendrocyte.

Six weeks after injury, quantification of BrdU^+^/s100β^+^ cells in the lvGFP control group showed that, as a fraction of BrdU^+^ cell density, 1.3 ± 0.3 % were identified as s100β^+^ astrocytes (**Figures 4A,C**). One-way ANOVA showed that the average fraction of BrdU^+^ cells co-labeled with s100β was not significantly different in the lvGDNF group (0.9 ± 0.3 %) but was significantly greater in animals treated with lvMeteorin (4.6 ± 1.5 %; *p* = 0.048, one-way ANOVA, Bonferroni *post hoc*; **Figure 4C**).

To look at the effects of treatment on oligodendrocyte lineage cells, cells were co-labeled for BrdU^+^ with the pan oligodendrocyte marker Olig2 and the post-mitotic oligodendrocyte marker APC. In all treatment groups we observed examples of BrdU^+^/Olig2^+^ cells that were both positive and negative for APC expression, indicative of oligodendrocyte progenitors (BrdU^+^/Olig2^+^/APC^-^) and more mature oligodendrocytes (BrdU^+^/Olig2^+^/APC^+^), respectively (**Figure 4B**). As a percentage of BrdU^+^ cell density, lvMeteorin significantly increased the fraction of Olig2^+^/APC^-^ oligodendrocyte progenitors (8.1 ± 0.8 %) compared to lvGFP-treated animals (3.3 ± 1%; *p* = 0.04, one-way ANOVA, Bonferroni *post hoc*), while there was no significant change between the lvGFP control group and the lvGDNF group (5.1 ± 2%) – **Figure 4D**. This equated to a significantly greater total number of BrdU^+^/Olig2^+^/APC^-^ oligodendrocyte progenitors in the anterior striatum of lvMeteorin treated animals (8.3 ± 0.8 × 10^3^ cells), compared to the lvGFP (3.1 ± 0.9 × 10^3^ cells) and lvGDNF groups (6.2 ± 2.2 × 10^3^ cells; lvGFP v lvMeteorin, *p* = 0.03, one-way ANOVA, Bonferroni *post hoc*).

We then assessed whether the Meteorin mediated increase in newborn oligodendrocyte progenitors translated to a greater increase in differentiation toward post-mitotic (APC^+^) oligodendrocytes. To do this, we assessed the fraction of BrdU^+^/Olig2^+^ cells that adopted an APC^+^ phenotype. In the lvGFP group, an average of 13.4 ± 4.3% of total BrdU^+^/Olig2^+^ cells co-expressed APC. Comparison between treatment groups revealed that there was no significant difference between lvGFP animals and lvMeteorin (8.0 ± 1.5 %) or lvGDNF animals (9.9 ± 3.1%; *p* = 0.71, lvGFP v lvMeteorin; one-way ANOVA, Bonferroni *post hoc*) – **Figure 4E**. This result was consistent when comparing the average total BrdU^+^/Olig2^+^/APC^+^ cell number between groups (n.s., *p* = 0.55, data not shown; lvGFP v lvMeteorin, one-way ANOVA, Bonferroni *post hoc*).

We assessed the effects of Meteorin treatment on microglia proliferation using the microglia marker Iba1 in conjunction with BrdU (**Figure 4F**). In the lvGFP treatment group, 36.8 ± 1.8% of BrdU^+^ cells were Iba1^+^, indicating that this is the predominant phenotype of newborn cells post-injury (**Figure 4G**). To determine whether Meteorin or GDNF treatment influenced microglia proliferation after striatal injury, Iba1/BrdU quantification was conducted across these groups. Compared to the lvGFP control group, no significant difference was observed in the percentage of Iba1^+^/BrdU^+^ cells in the lvMeteorin groups (49.3 ± 8.2% of BrdU^+^ cells; *p* = 0.27) and the lvGDNF group (31.1 ± 5% of BrdU^+^ cells; *p* = 0.72; one-way ANOVA, Bonferroni *post hoc*) – **Figure 4G** indicating that these factors did not influence microglia proliferation post-striatal injury.

## Discussion

These results show that lentiviral delivery of Meteorin to the damaged striatum leads to robust over-expression of the diffusible protein resulting in increased gliogenesis but not neurogenesis in response to striatal injury. Cell proliferation is a well-established response to acute brain injury (Cavanagh, 1970; Nait-Oumesmar et al., 1999) and it has been convincingly demonstrated that the majority of the newborn cells are local microglia (Marty et al., 1991; Amat et al., 1996) which we have shown accounts for 40% of newborn cells post-injury. Importantly, it has also been shown that, following damage to the striatum, a small proportion of the newborn cells at the site of injury are neurons that migrate from the adjacent SVZ (Arvidsson et al., 2001; Parent et al., 2002). This has led to the idea that augmentation of this neurogenic response might lead to regenerative therapies for brain repair.

A number of studies have significantly improved the level of injury-induced striatal neurogenesis through delivery of growth factors, either as protein infusions (Pencea et al., 2001; Kobayashi et al., 2006; Ninomiya et al., 2006) or using viral vectors (Chmielnicki et al., 2004; Benraiss et al., 2012; Yu et al., 2013). Here we chose to investigate Meteorin in this context based on previous work showing that it is up-regulated in the striatum following injury (Jorgensen et al., 2011; Wang et al., 2012; Lee et al., 2015), can protect striatal neurons from excitotoxic death (Jorgensen et al., 2011) and is a potent chemokinetic regulator of migratory doublecortin^+^ neuroblasts in the striatum after middle cerebral artery occlusion (MCAO) (Wang et al., 2012). Although immunohistochemical analysis showed robust and diffuse over-expression of Meteorin throughout the lesioned striatum, there was no significant impact on the number of new striatal neurons generated in the first week after injury relative to the lvGFP control group. This is in contrast to a similar study recently reporting that Meteorin infused as a protein over 2 weeks following medial cerebral artery occlusion significantly increases the number of newborn neurons in the striatum by around 70% (Wang et al., 2012). The conflicting findings might be at least partly explained by experimental variables, including differences in the timing and dose of Meteorin delivery and also the severity of striatal damage. Notably, however, over-expression of GDNF using the same lentiviral construct delivered at the same time relative to injury resulted in a significant increase in the number of new neurons compared to the Meteorin and control groups.

We included a GDNF group in order to contextualize the impact of Meteorin. GDNF is well established as a neurotrophic factor in models of CNS injury and has previously been shown to increase the number of newborn cells that survive as mature neurons in the striatum after striatal injury (Kobayashi et al., 2006). We observed an 11-fold increase in the number of mature striatal neurons after striatal injury in GDNF treated animals, but no significant difference in Meteorin treated animals under the same experimental conditions. Collectively, the results suggest that the neurogenic impact of Meteorin following striatal damage is modest and may be relatively more sensitive to factors related to timing and dose as well as the nature of the injury. Understanding the efficacy of neurotrophic proteins in specific injury models is an important challenge for developing regenerative gene-based therapies for CNS injury.

Another potential role for neurotrophic factors in modulating the neurogenic response to injury is through impact on the differentiation of newborn cells toward therapeutically relevant cell types — for example striatal projection neurons that can replace those lost after damage. Based on immunohistochemistry for Darpp32 6 weeks after lesioning, no examples of new striatal projection neurons were found across any of the three treatment groups. However, a number of BrdU^+^/NeuN^+^ cells were found to acquire a Calretinin^+^ interneuron phenotype. These findings are consistent with a recent study showing that striatal injury can facilitate migration of nearby SVZ neuroblasts to the site of injury but does not influence their original, interneuron differentiation potential (De Marchis et al., 2007). Neither GDNF nor Meteorin influenced the fraction of BrdU^+^/NeuN^+^ cells to adopt a Calretinin^+^ identity. Thus the mechanism underlying greater neuronal numbers in the GDNF group is likely to be related in increased survival rather than an influence on differentiation potential. We also observed many BrdU^+^/Calretinin^+^ cells that did not express NeuN. This may represent a different Calretinin^+^ phenotype or reflect an immature status of these cells, as has been shown in studies on staging of protein expression in hippocampal neurogenesis (Brandt et al., 2003). It is important to note that the majority of BrdU^+^/NeuN^+^ cells did not express Calretinin or Darpp32. This may reflect an immature phenotype within the newborn population of striatal neurons. However, previous studies have shown that insult-mediated neurogenesis in the striatum produces other interneuron phenotypes characterized by the markers parvalbumin and neuropeptide Y (Collin et al., 2005) which may also account for the remaining newborn neurons.

The most prominent impact of Meteorin over-expression on newborn cells generated after injury was a significant increase in the proportion of glial cell types, including s100β^+^ mature astrocytes. This is consistent with previous *in vitro* findings using primary cultures, where Meteorin has been shown to act through the Jak-STAT3 pathway to increase astrocyte differentiation (Nishino et al., 2004; Lee et al., 2010; Sun et al., 2014). We have previously reported that Meteorin is up-regulated in s100β cells at the site of injury after excitotoxic lesioning of the striatum. Taken together, these data suggest that Meteorin may act as a paracrine signaling molecule to drive gliosis via differentiation in response to brain damage as opposed to acting as a trophic factor for these cells. We also looked at microglial phenotype amongst newborn cells. Iba1+ microglia accounted for ~40% of all newborn BrdU^+^ cells post injury. This is in line with previous literature which describes an early and rapid proliferative response of microglia after acute injury in the CNS (Morioka et al., 1993; Topper et al., 1993; Thored et al., 2009; Taylor and Sansing, 2013). The lack of impact on the proportion of BrdU^+^ microglial cells suggests a specific function for Meteorin in astrocyte differentiation rather than a broad exacerbation of inflammatory response to injury.

Interestingly, Meteorin also increased the number of oligodendrocyte progenitor cells (OPCs). Here we report that Meteorin significantly increases the number of Olig2^+^ OPCs in the striatum following excitotoxic damage. This reveals a novel biological function for Meteorin *in vivo* and indicates therapeutic potential in pathological settings involving loss of oligodendrocytes—for example the demyelination of neurons resulting from loss of oligodendrocytes after ischemic stroke (Pantoni et al., 1996; Dewar et al., 2003). Notably, while the OPC pool increased in Meteorin treated animals, this did not translate to an increase in mature APC^+^ oligodendrocytes. This may reflect that additional signaling components are required for maturation in the lesioned striatum and/or an unfavorable environment for differentiation. In future studies it will be valuable to assess and compare the oligogenic effects of Meteorin with other molecules reported to drive oligodendrogliogenesis after injury, such as Erythropoietin (Zhang et al., 2010) and Noggin (Irvin et al., 2008) as well as the mechanism driving this increase in OPCs to determine whether Meteorin influences the proliferation and differentiation of this population or offers trophic support to OPCs. Assessment of the timing and combinatorial delivery of these molecules may reveal an optimal approach for increasing both the number of oligodendrocytes and their capacity for myelination following de-myelinating injuries. Other valuable future studies to extend on the present findings would include lineage tracing work aimed at identifying the cells that respond to MTRN in order to drive gliogenesis. There is strong evidence to suggest that the neurogenic response in a variety of striatal injury models, including MCAO (Arvidsson et al., 2002), QA lesioning (Collin et al., 2005), and traumatic brain injury (Richardson et al., 2007) is largely provided by the nearby pool of neurogenic precursors in the SVZ. The origin of the gliogenic response is less well characterized but may include both an SVZ component – for example OPCs can be generated by SVZ precursors and migrate into the striatum following stroke (Li et al., 2010) – as well as the proliferation of local parenchymal progenitors or differentiated glia at the site of injury.

In summary, we demonstrated that Meteorin was able to increase astrogenesis in the striatum after acute injury characterized by widespread neuronal cell loss. We also revealed a novel function for Meteorin in significantly increasing the number of OPCs post-injury. Interestingly, Meteorin did not influence the maturation of these cells into mature oligodendrocytes, suggesting a discrete function on the OPC population. Meteorin was not effective for augmentation of the neurogenic response to injury. These findings highlight that development of strategies for brain-repair based on trophic-factor delivery will likely require combinatorial approaches, where specific proteins are targeted to affect the survival and differentiation of specific cell types.

## Author Contributions

JW, LT, and CP performed surgical procedures. JW processed, imaged, and quantified the tissue obtained. CE acquired representative images and JJ constructed the lentiviral vectors for gene delivery. LT funded and contributed to the design and analysis of the study.

## Conflict of Interest Statement

The authors declare that the research was conducted in the absence of any commercial or financial relationships that could be construed as a potential conflict of interest.
